# Ultrasound Doppler findings of a Twin Reversed Arterial Perfusion Sequence (TRAP)

**DOI:** 10.12669/pjms.40.1.8088

**Published:** 2024

**Authors:** Saba,a Abdulmalik Amer, Sultan Abdulwadoud Alshoabi, Abdulaziz A. Qurashi, Fahad H. Alhazmi, Rawda Yehia Al-Rudini

**Affiliations:** 1 Saba’a Abdulmalik Amer Department of Radiology, University of Science and Technology Hospital (USTH), Radiology Department, College of Medicine, 21 September University of Medical and Applied Sciences, Sana’a, Republic of Yemen; 2 Sultan Abdulwadoud Alshoabi Department of Diagnostic Radiology Technology, Department of Diagnostic Radiology Technology, College of Applied Medical Sciences, Taibah University, Almadinah Almunawwarah, Kingdom of Saudi Arabia; 3 Abdulaziz A. Qurashi Department of Diagnostic Radiology Technology, Department of Diagnostic Radiology Technology, College of Applied Medical Sciences, Taibah University, Almadinah Almunawwarah, Kingdom of Saudi Arabia; 4 Fahad H. Alhazmi Department of Diagnostic Radiology Technology, Department of Diagnostic Radiology Technology, College of Applied Medical Sciences, Taibah University, Almadinah Almunawwarah, Kingdom of Saudi Arabia; 5 Rawda Yehia Al-Rudini Department of Radiology, Al-Thawra Modern General Hospital, Sana’a, Republic of Yemen

**Keywords:** Twin reversed arterial perfusion sequence, Monochorionic twin (share a single placenta), Monoamniotic twin (share an amniotic sac). Acardiac fetus, Pump fetus

## Abstract

Twin reversed arterial perfusion (TRAP) sequence is a rare pregnancy complication occurs in an identical twin pregnancy that share a single placenta. TRAP sequence is a twin’s pregnancy includes a normal-developed viable pump fetus and an abnormal usually nonviable acardiac fetus. The nonviable acardiac fetus depends on the pump fetus for his blood supply and put the pump fetus at risk of high cardiac output heart failure and congenital anomalies with high mortality rate. Gray-scale ultrasound and color and pulsed Doppler imaging is a noninvasive accessible imaging modality for the diagnosis of TRAP sequence.

Early diagnosis of such conditions is mandatory in order to apply the proper therapeutic measures and to help the normal developed pump fetus to survive. The main goal of management is to interrupt blood supply to the nonviable acardiac fetus to reduce the strain on the heart of the pump fetus thus, increase the chance of survival.

## INTRODUCTION

Twin reversed arterial perfusion (TRAP) sequence is a rare pregnancy complication occurs in an identical twin pregnancy that share a single placenta (monochorionic). The pregnancy has a vascular anastomosis connects the circulation of both fetuses with a coexistent of an abnormal acaridac twin and another normal-developed pump twin. The abnormal acardiac twin is a parasite dependent upon the pump twin that provide blood circulation by vascular anastomosis and puts the pump fetus at risk of high cardiac output failure.^1^,^2^ The acardiac twin is a rare complication of monozygotic twin pregnancy that occurs in one of every 35,000 twin pregnancies and in 1% of monochromatic twin pregnancies. The majority of pump twins are normal but cardiogenic defects, gastroschisis, and musculoskeletal anomalies may observe with approximately 50% reported mortality rate.^2^,^3^ Obstetric ultrasound imaging can diagnose TRAP syndrome, and Doppler examination confirms the reversed direction blood flow in the acardiac twin.^4^,^5^

The aim of this manuscript is to document a rare case of TRAP twin (acardiac fetus) with a total absence of the cranium. The case was discovered in the 26^th^ week of gestation who was delivered non-viable, and anencephalic with deformed upper body at the 30^th^ week of gestation. This case report elucidates the importance of the gray-scale ultrasound and color and pulsed Doppler imaging as a highly valuable tool in diagnosing such cases early, in detecting of poor prognostic features and in following up TRAP sequence. The report will be significant for the obstetricians, sonographers, and radiologists who should be aware regarding the diagnosis of this congenital anomaly in twin pregnancy with the consequent results of early diagnosis of such conditions to take the proper therapeutic measures and to survive the normal pump twin.

## CASE PRESENTATION

A 37-year-old woman, Gravida-5, Para 3+0, presented at the 6^th^ month of pregnancy complaining of large for date uterus with dyspnea. She had no history of consanguinity, previous pregnancies with congenital anomalies, chronic medical diseases, nor drug abuse. Clinical examination showed pregnant female with large-for-date uterus and fundal level reaching up to epigastric region.

**Fig.1:**
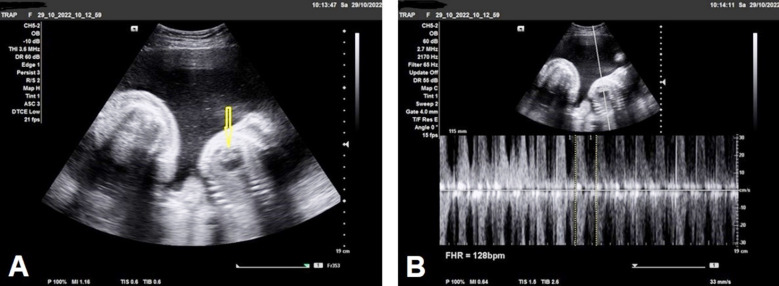
**(A)** Gray-scale ultrasound study shows two intrauterine fetuses in a single amniotic cavity, the right twin appears structurally normal-developed with no gross congenital (pump fetus), (arrow). The left twin appears structurally abnormal with no visible cardiac tissue or activity (acardiac fetus). **(B)** Doppler ultrasound shows two intrauterine fetuses in a single amniotic cavity, the right fetus shows positive regular cardiac activity.

Gray-scale ultrasound imaging showed two intra-uterine fetuses with a single fundo-anterior placenta, and single amniotic cavity (monoamniotic) with heart activity in the first fetus (pump twin) and no visible heart activity in the second fetus (acardiac twin), (Fig.1A). Color and Pulsed Doppler showed pulsating heart in the pump fetus (Fig.1B).

**Fig.2:**
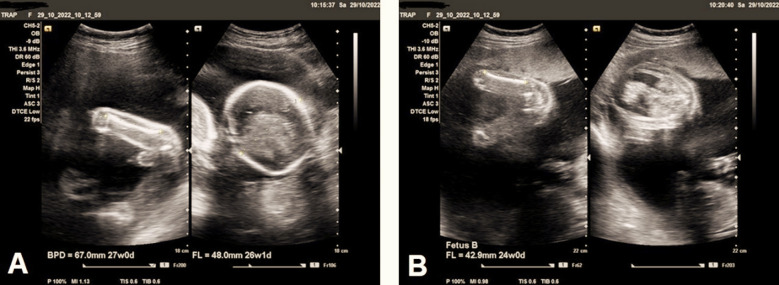
**(A)** Ultrasound imaging study shows the gestational age of the pump twin as 27 weeks by biparietal diameter (BPD), and 26 weeks+1 day by femur length (FL), and 27 weeks by abdominal circumference (AC), with about 983 gram expected fetal weight. The pump fetus appears structurally normal with no gross congenital anomalies with no signs of hydrops. **(B)** Ultrasound imaging study of the acardiac twin which was stuck to the anterior uterine superiorly by amniotic band and shows markedly under-developed upper body organs including upper limbs, upper chest, non-developed head, and non-developed heart, with around 24 weeks gestational age by femur length (FL).

**Fig.3:**
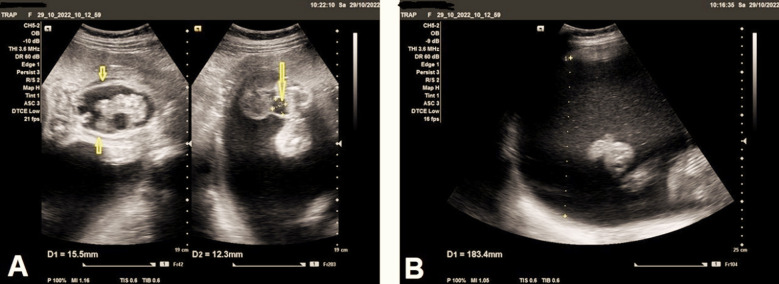
**(A)** Ultrasound imaging study of the acardiac twin which was stuck to the anterior uterine superiorly by amniotic band and shows markedly under-developed upper body organs including upper limbs, upper chest, non-developed head, and non-developed fetal heart. In addition to marked ascites, and edematous changes of the fetal body (signs of fetal hydrops), (short arrows), and cyst in the base of the umbilical cord of about 12mm in maximal dimension (long arrow). **(B)** Ultrasound imaging study shows markedly increased amniotic fluid volume, the largest pocket of approximately 183 mm, and amniotic fluid index (AFI) around 30 cm, indicating marked polyhydramnios.

The pump fetus developed in normal structure with normal cardiac morphology and regular cardiac activity, without gross congenital anomalies. No signs of heart failure or fetal hydrops were noted with average gestational age (GA) of 27 weeks by biparietal diameter (BPD), and abdominal circumference (AC), and 26 weeks+1 day by femur length (FL), with about 983 gram expected fetal weight (Fig.2A). The acardiac twin was stuck to the anterior uterine superiorly by amniotic band and showed markedly under-developed upper body organs including upper limbs, upper chest, non-developed head, and non-developed heart, with around 24 weeks gestational age by FL (Fig.2B), in addition to marked abdominal ascites, and marked edematous changes of the body as signs of fetal hydrops (Fig.3A).

**Fig.4:**
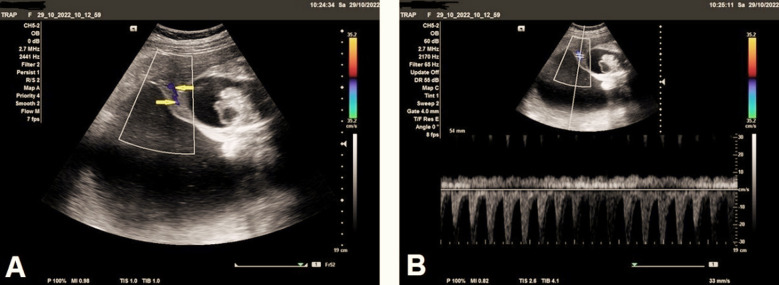
**(A)** Color Doppler examination shows acardiac twin with short umbilical cord containing only two-vessels (arrows), indicating single umbilical artery, and **(B)** Ultrasound Doppler examination shows acardiac twin with short umbilical cord containing only two-vessels, indicating single umbilical artery with reverse blood flow.

**Fig.5:**
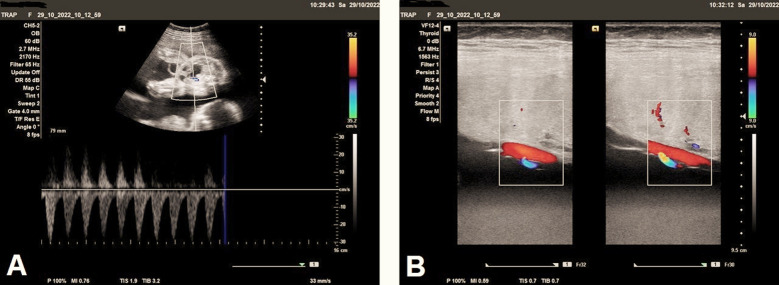
**(A)** Color and spectral Doppler examination shows acardiac twin with reversed direction of vascularity seen in iliac blood vessels. **(B)** Color and spectral Doppler examination shows anastomotic superficial blood vessels seen crossing the placental surface connecting the short umbilical cord vasculature of the acardiac twin to the pump twin.

The uterus showed markedly increased amniotic fluid volume, the largest pocket of approximately was 18.3 cm, and the amniotic fluid index (AFI) around 30 cm, indicating marked polyhydramnios (Fig.3B). Color and pulsed Doppler examination showed structurally abnormal acardiac fetus with no heart activity, and short two-vessels of the umbilical cord (Fig.4A) indicating single umbilical artery (Fig.4B), with reversed direction of blood flow seen in iliac blood vessels (Fig.5A). Anastomotic superficial blood vessels were seen crossing the placental surface connecting the short umbilical cord vasculature of acardiac twin to the pump twin (Fig.5B).

### Follow up and outcome

At 30 weeks of gestation, the patient experienced sudden premature delivery with the following outcome of two fetuses: The pump twin survived for about 10 minutes then expired. The acardiac twin was delivered non-viable, anencephaly with deformed upper body. The placenta was retained and the mother patient suffered from severe vaginal bleeding leading her urgently underwent operative removal of placenta with compensatory blood transfusion. Final diagnosis was monochorionic monoamniotic twins’ pregnancy with TRAP syndrome and marked polyhydramnios.

## DISCUSSION

TRAP sequence is a rare complication occurs exclusively in an identical twin pregnancy sharing a single placenta (monochorionic) with vascular anastomoses connecting the circulation of both fetuses. In TRAP syndrome, the twin’s pregnancy includes a normal-developed (pump) fetus and an abnormal (acardiac) fetus who depends on the pump foetus to provide blood circulation by vascular anastomosis thus putting the pump fetus at risk of high cardiac output heart failure and congenital anomalies with approximately 50% reported mortality rate for the pump fetus.^1^-^3^

In this manuscript very rare case of monochorionic monoamniotic twins’ pregnancy with TRAP syndrome and marked polyhydramnios was reported. Monochorionic twins occurs when a single egg (zygote) is fertilized and divides into two separate twins (monozygotic) who share the same genes (identical genotype), however they may report discordant phenotype.^6^ Monoamniotic (share an amniotic sac) twins are a twin pregnancy with a single amniotic sac with a single placenta and accounts for 1% - 5% of monozygotic pregnancies.^7^ The cause of TRAP sequence in the acardiac twin is the development of arterio-arterial vascular anastomosis between the umbilical arteries of the identical monozygotic twins during embryogenesis.

The acardiac twin lack direct vascular connection with the placenta, and the blood goes into the acardiac twin via the umbilical artery and leaves via the umbilical vein in totally opposite blood flow directions from the normal placenta-fetal circulation.^8^ Our patient presented with typical case of TRAP sequence in which, color and pulsed doppler study showed structurally abnormal acardiac fetus with short umbilical vessels, with reversed direction of vascularity in iliac blood vessels, and anastomotic superficial blood vessels seen crossing the placental surface connecting the short umbilical cord vasculature of the acardiac fetus to the pump fetus .

In the current case, the pregnancy was complicated with polyhydramnios and the output was premature delivery at 30^th^ week of gestation. The pump fetus survived for only 10 minutes then expired, while the acardiac fetus was delivered non-viable and anencephaly with deformed upper body. These results are consistent with a previous case report by Buyukkaya who reported that, the mortality rate is 100% in the acardiac fetus, and around 50% in the pump fetus usually attributed to cardiac output heart failure and sometimes due to prematurity caused by polyhydramnios.^9^ The prognosis is related to the weight of the recipient acardiac twin.

The higher body wight of the acardiac twin increase the blood supply demand, which increases the chance of developing of cardiac insufficiency in the pump twin.^10^ The weight of the acardiac fetus could not be calculated by measuring the biometric parameters of the head circumference, AC, and FL using the standard formula (such as Hadlock^’^s). However, weight of the acardiac fetus can be calculated using the following formula:

**(1.2 × longest length^2^) – (1.7 × longest length).^11^**

Doppler velocimetry findings including resistive index (RI) of the umbilical arteries can be a prognostic factor for pump fetus. If the difference in RI between the pump fetus and the acardiac fetus is > 0.2, the prognosis will be good. However, smaller differences in RI are associated with poor outcomes of the pump fetus due to cardiac failure and hypoperfusion of the central nervous system.^12^

The main goal of managing TRAP sequence is to help the viable pump fetus to survive and to reach the term for delivery of a normal newborn.^2^ Conservative management with close monitoring is a safe option for managing TRAP sequence when the weight of the acardiac fetus is ≤ 50% the weight of the pump fetus.^13^ Other management options include prenatal occlusion of blood flow to the nonviable acardiac fetus by fetoscopic ligation, laser coagulation of the umbilical cord, bipolar cord cauterization, or ablation using radiofrequency.^10^ Fetoscopic laser photocoagulation of the communicating vessels of the placenta, in particular transection of the umbilical cord of the TRAP seems to be a useful treatment method for TRAP.^14^ Bipolar cord coagulation to interrupt blood perfusion to the non-viable acardiac fetus through amnio patch with autologous platelet concentrate followed by cryoprecipitate amnioinfusion was reported as an effective procedure to manage TRAP sequence.^15^ More treatment options could be valuable for further research in the future.

## CONCLUSION

Gray-scale ultrasound and Doppler imaging is a noninvasive accessible imaging modality for the diagnosis of TRAP sequence, which is a twin’s pregnancy includes a normal-developed viable pump fetus and an abnormal nonviable acardiac fetus. The nonviable acardiac fetus depends on the pump fetus or his blood supply and putting the pump fetus at risk of high cardiac output heart failure and congenital anomalies with high mortality rate. Early diagnosis of such conditions is mandatory in order to apply the proper therapeutic measures and to help the normal developed pump fetus to survive. The main goal of management is to interrupt blood supply to the nonviable acardiac fetus to reduce the strain on the heart of the pump fetus thus, increase the chance of survival.

### Author’s Contribution:

**SAA (Saba Abdulmalik Amer) & RYA:** Provided ultrasound Doppler exam and data collection. **SAA (Sultan Abdulwadoud Alshoabi):** Wrote the manuscript.

**AAQ & FHA:** Revised the manuscript and improved language.

**SAA (Saba Abdulmalik Amer) & SAA (Sultan Abdulwadoud Alshoabi)** are responsible for the contents and integrity of the article.
